# Re-validation of the Kenny Music Performance Anxiety Inventory-revised among Chinese university students majoring in vocal music

**DOI:** 10.3389/fpsyg.2025.1667404

**Published:** 2025-09-26

**Authors:** Li Pan, Qihuan Chen, Lan Lou, Qizhen Gu, Huiqin Luo, Ping Li, Yannan Liu

**Affiliations:** ^1^School of Humanities, Arts, and Education, Shandong Xiehe University, Jinan, China; ^2^School of Education and Foreign Languages, Wuhan Donghu University, Wuhan, China; ^3^Graduate School of Business and Advanced Technology Management, Assumption University, Bangkok, Thailand

**Keywords:** Chinese students, confirmatory factor analysis, exploratory factor analysis, music performance anxiety, vocal music

## Abstract

**Introduction:**

Due to its significant impact on performers’ psychological well-being and career development, music performance anxiety (MPA) has recently received growing public attention. The Kenny Music Performance Anxiety Inventory-Revised (K-MPAI-R) is one of the most widely focused MPA scales, often used to assess the manifestations and influencing factors of MPA in musicians across different genres and professional stages. However, most current K-MPAI-R validation studies did not distinguish between vocal and instrumental performers. As they differ significantly in performance settings and stressors, these variations may impact the validity of the scale and lead to biased results.

**Methods:**

With Kenny’s permission, this study aims to re-examine the reliability and validity of the K-MPAI-R in the context of Chinese university students majoring in vocal music. 736 Chinese students participated in this study. The researchers divided the total sample into two equal subsamples, each consisting of 368 students. One subsample was subjected to exploratory factor analysis (EFA) and the other to confirmatory factor analysis (CFA).

**Results:**

Through EFA, the researchers obtained a four-factor model (F1: Psychological Vulnerability, F2: Proximal Somatic and Cognitive Anxiety, F3: Parental Support, F4: Memory Self-Efficacy); subsequently, CFA was employed to confirm that the model demonstrated a good fit and exhibited adequate reliability and validity.

**Discussion:**

This study represents the first refined validation of the K-MPAI-R scale for Chinese university students majoring in vocal music, providing cross-cultural and discipline-specific evidence for optimizing measurement tools to assess MPA among vocal performers accurately.

## 1 Introduction

Music Performance Anxiety (MPA) is frequently characterized as a complicated, multi-faceted emotional reaction and a prevalent psychological condition. MPA generally occurs during public performances, characterized by various cognitive concerns, physiological arousal, and behavioral avoidance elicited by the expectation of or the act of performing (Kenny, 2011). MPA is context-dependent, mainly arising from evaluative performance situations, and is frequently linked to perceptions of others’ evaluations, fear of failure, and elevated expectations of musical excellence (Papageorgi et al., 2007). Simultaneously, MPA significantly impacts various music performers, especially among university music majors, frequently resulting in diminished performance, reduced self-confidence, and potential reevaluation of career paths (Driskill, 2012; Fernholz et al., 2019). Consequently, MPA has been identified as a significant risk factor influencing musicians’ mental health and career sustainability (Fernholz et al., 2019; Kenny, 2011).

Numerous scholars have developed assessment tools for MPA, among which the Kenny Music Performance Anxiety Inventory-Revised (K-MPAI-R) has garnered the most attention in recent years. The K-MPAI-R emphasizes that MPA arises not only from the pressures of performance contexts but, more significantly, is intricately linked to individuals’ early attachment patterns, self-esteem, and internal psychological conflicts (Kenny, 2011). The K-MPAI-R evaluates the emotional, physiological, cognitive, and behavioral aspects of performance anxiety while also seeking to uncover the underlying psychological developmental pathways (Kenny, 2011). Thus far, the K-MPAI-R has been validated across diverse contexts, with varied research methodologies applied to different study populations. However, research findings indicate significant discrepancies in its structural model, factor division, and item attribution (Philippe et al., 2022; Barros et al., 2024; Chang-Arana et al., 2018; Dias et al., 2022). All the aforementioned studies were conducted within unique cultural contexts, and the discrepancies in their findings may stem from differences in individuals’ cognitive patterns toward anxiety and emotional expression habits across various cultures. Consequently, it is essential to further validate the structural validity and interpretive capacity of the scale across different cultural contexts to ensure its cross-cultural applicability.

Culture plays a central regulatory role in shaping individuals’ emotional experiences, expression styles, and their social acceptability (Matsumoto et al., 2008). Western cultures, represented by the United States, Australia, and Europe, typically encourage individuals to express their inner emotions, especially negative ones (Hofstede et al., 2010; Matsumoto et al., 2008). By contrast, East Asian cultures, particularly those in Confucian tradition-based societies, such as China, place a greater emphasis on social norms, including interpersonal harmony, “face” culture, and emotional suppression, and are less inclined to express negative emotions like MPA (Han, 2016; Huang, 2016). The Confucian concept of “interpersonal harmony” prompts individuals to pay heightened attention to others’ feelings and evaluations during public performances, with music performers being prone to excessive evaluative concern stemming from worries about “whether they meet others’ expectations” (Matsumoto et al., 2008). Furthermore, “face” culture emphasizes the preservation of individuals’ and families’ social images, leading performers to directly associate mistakes in their performances with the shame of “losing face,” thereby intensifying fear of failure and self-denial (Zane and Yeh, 2002). Moreover, the notion of emotional suppression leads individuals to tend to conceal their authentic emotional experiences, particularly negative ones such as anxiety, shame, and unease, as these emotions are perceived to disrupt interpersonal relationships or induce social stigma (Hofstede et al., 2010; Markus and Kitayama, 2014). However, the majority of current MPA studies have been conducted within Western cultural contexts. This study, focusing on Chinese music-major undergraduates under the influence of Confucian culture, holds significant importance for revealing the specific manifestations of MPA in Confucian cultural contexts and enhancing the cross-cultural validity of assessment tools.

Furthermore, the differences in the perception of MPA among individuals across various music performance forms should not be overlooked by researchers. Notably, due to fundamental differences in their performance modalities, vocal and instrumental performers exhibit distinct patterns in terms of how performance environments trigger anxiety and how they experience psychological pressure (Ryan and Andrews, 2009). Kenny (2011) points out that instrumentalists can “recede” behind their instruments, and their performances prioritize technical execution over self-presentation, which diminishes the stressful experiences arising from emotional exposure. In contrast, vocal performers, who directly use their bodies as instruments, have their voices, images, and even facial expressions directly perceived by the audience, making them more prone to heightened self-awareness and evaluative apprehension (Kenny, 2011). This high-exposure performance characteristic often subjects vocal performers to greater psychological pressure when taking the stage, and their ways of perceiving MPA as well as the intensity of such perception differ significantly from those of instrumentalists (Kenny, 2011). To date, MPA-related studies have not distinguished between vocal performers and instrumentalists, which may lead to an insufficiently precise understanding of the MPA characteristics of these two groups of performers and undermine the applicability of research findings across different performance populations.

Therefore, this study aims to: (1) re-validate the reliability, validity, and structural construct of the K-MPAI-R among Chinese undergraduate students majoring in vocal music, a group whose values are shaped by Confucian cultural norms; (2) explore the underlying reasons for cross-cultural differences in MPA by comparing the validated structure of the current Chinese sample with findings from K-MPAI-R validation studies in Western and other East Asian contexts; and (3) analyze differences in MPA characteristics between the current pure vocal sample and other types of samples (instrumental or mixed samples) reported in previous studies, particularly focusing on variations in anxiety triggers and expressions related to the “instrumental shielding” effect.

## 2 Literature reviews

### 2.1 Music Performance Anxiety (MPA)

Music Performance Anxiety (MPA) is the experience of persistent, distressing nervousness and worry in public music performances, accompanied by noticeable deterioration in performance skills that is disproportionate to one’s musical ability, training, or preparation, even in talented, well-practiced, and fully prepared performers (Salmon, 1990). Kenny (2009) described Music Performance Anxiety (MPA) as a complex, multifaceted phenomenon, defining it as significant and persistent anxious worry related to music performance. This anxiety stems from specific anxiety-conditioned experiences and manifests as a combination of emotional, cognitive, physiological, and behavioral symptoms.

Music Performance Anxiety manifests symptoms across physiological, emotional, behavioral, and cognitive domains. At the physiological level, music performers experiencing high levels of MPA may exhibit symptoms such as rapid breathing, trembling hands and feet, sweating, dry mouth, and accelerated heartbeat. These reactions represent the body’s instinctive stress response to potential threats, reflecting the overactivation of the autonomic nervous system (Kenny, 2023). At the emotional level, MPA primarily manifests as pre-performance worry and tension, as well as fear and anxiety during performances (Bascomb, 2019; Spahn, 2015). Such emotional fluctuations not only affect performers’ psychological states but may also further exacerbate physiological discomfort (Spahn, 2015). At the behavioral level, MPA may lead to overt avoidance behaviors (e.g., avoiding performing or practicing), covert avoidance behaviors (e.g., avoiding eye contact or refraining from playing technically demanding pieces), and loss of technical control (Juncos et al., 2017; Salmon, 1990). It may even result in extreme behaviors such as over-learning, excessive preparation, or self-medication (Spahn, 2015). At the cognitive level, MPA manifests as memory impairment, inattentiveness, and excessive focus on perceived threats (such as personal evaluations of one’s performance and audience feedback). These cognitive biases further exacerbate performers’ anxiety, hindering their ability to perform at their usual level (Faur et al., 2021; Steptoe, 2001). The symptoms of MPA in these four categories are independent of each other (Spahn, 2015).

### 2.2 Development and revision of K-MPAI-R

Kenny et al. (2004) initially developed the 26-item Kenny Music Performance Anxiety Inventory (K-MPAI) to assess factors related to music performance anxiety (MPA). However, the original scale had certain limitations, and it may not have fully captured the multifaceted nature of the anxiety experience. To overcome this limitation, Kenny (2009) updated the MPA framework based on Barlow (2000) emotional theory of anxiety disorders and revised the scale (K-MPAI), expanding the number of items to 40, forming the latest version of the Kenny Music Performance Anxiety Inventory-Revised (K-MPAI-R). The revised scale not only assesses the performer’s potential psychological vulnerability but also considers pre-performance experiences, making the measurement of MPA more comprehensive and providing a broader therapeutic focus for performing artists suffering from performance anxiety.

Kenny (2009) conducted an in-depth validity analysis of the K-MPAI-R. This study sampled 151 undergraduate students majoring in music and dance from the University of Auckland, with 72% being music majors and 28% dance majors. Using Principal Components Analysis with varimax rotation for exploratory factor analysis (EFA), three core factors were extracted: Early Relationship Context (6 items), Psychological Vulnerability (12 items), and Proximal Performance Concerns (22 items). The K-MPAI-R quickly became one of the classic tools for assessing MPA once it was introduced.

### 2.3 Revalidation of the K-MPAI-R across cultures and regions

With the widespread application of the K-MPAI-R, an increasing number of researchers have begun to re-examine its structural stability and applicability across different cultural (national) contexts. However, most studies conducted in diverse cultural settings have yielded considerably varied results, including differences in the number of extracted factors, the number of items corresponding to each factor, and the specific factors to which individual items align. It is also worth noting that the EFA methods employed in these studies, including approaches to factor extraction and rotation, exhibit significant variations, which constitutes one of the reasons for the discrepancies above.

Barbar et al. (2015) validated the Brazilian Portuguese version of the scale using a sample of 230 adult musicians (41.6% of whom were primarily vocalists, 18% were string instrument players, and 10.8% were keyboard instrument players). They conducted an EFA using Principal Components Analysis with Varimax Rotation. The initial model explained 62.4% of the variance, and after model screening, three factors were finally identified: Worries and Insecurity (10 items), Depression and Hopelessness (9 items), and Early Parental Relationships (4 items).

Alzugaray et al. (2016) validated the Spanish version of the scale, analyzing a sample of 490 instrumentalist musicians from music conservatories using both EFA and Confirmatory Factor Analysis (CFA). For EFA, Principal Components Analysis with Oblimin rotation was applied. Three factors were extracted: Specific Cognitions (11 items), Helplessness (10 items), and Early Family Context (4 items), with the initial EFA model accounting for 58.26% of the variance. Additionally, the research team used CFA to examine the fit of the proposed three-factor model, confirming that the new structural model demonstrated good fit.

Chang-Arana et al. (2018) further validated the Spanish version of the scale. Their study employed High Order Exploratory Factor Analysis (HOEFA), utilizing the Unweighted Least Squares (ULS) extraction method, promax oblique rotation, and the Schmid–Leiman Solution (SLS) orthogonal transformation. The analysis was conducted on two samples: 455 music performance majors from Peruvian higher music education institutions and 368 professional orchestral musicians from Australia. One higher-order factor (Negative Affectivity about Music Performance Anxiety) and two first-order factors were extracted. The first-order factors were Music Performance Anxiety (21 items for the Peruvian sample and 22 items for the Australian sample) and Depression (10 items for the Peruvian sample and 13 items for the Australian sample). The initial EFA model explained 41.17% of the variance in the Peruvian sample and 47.89% in the Australian sample, while the higher-order factor accounted for 58.65% and 43.98% of the variance, respectively. Additionally, the three-factor model demonstrated good structural similarity and invariance across the two samples, thus validating the consistent structure of the K-MPAI across different cultural contexts.

Faur et al. (2021) validated the Romanian version of the scale. They conducted an EFA using Principal Axis Factoring with Oblimin rotation, analyzing a sample of 420 Romanian musicians (42.19% string players, 11.66% woodwind players, 24.04% percussionists, 2.85% brass players, 15.95% vocalists, and 4.28% others). Four factors were extracted: Music Performance Anxiety Symptoms (18 items), Parental Support (3 items), Depression and Hopelessness (7 items), and Memory Self-Efficacy (2 items). The initial 8-factor EFA model explained 49.16% of the variance, while the final 4-factor model accounted for 41.37% of the variance.

Philippe et al. (2022) validated the French version of the scale. They employed EFA using Principal-Axis Factoring with Varimax rotation, higher-order EFA with minimum residual extraction and Oblimin rotation, and CFA. The analysis was conducted on a sample of 211 music students from various music schools and conservatories in the French-speaking region of Switzerland (mean age 25.34 years, 55% female). Five factors were extracted: Proximal Somatic and Cognitive Anxiety (11 items), Self/Other Scrutiny and Evaluation (6 items), Psychological Vulnerability (9 items), Confidence in Memory (2 items), and Early Parental Relationship Context (2 items). The initial first-order EFA model explained 39.3% of the variance, while the higher-order model accounted for 41.5% of the variance. Subsequently, they used CFA again to confirm that the proposed five-factor model exhibited good fit.

Dias et al. (2022) validated the Portuguese version of the scale using EFA with Principal Components Analysis and Varimax rotation. The analysis was conducted on a sample of 164 Portuguese undergraduate music students, comprising 148 instrumentalists (90.2%) and 15 vocalists (9.1%). Four factors were extracted: MPA-Related Symptoms (18 items), Depression and Hopelessness (7 items), Parental Support (3 items), and Memory Self-Efficacy (2 items). The initial EFA model explained 50.63% of the variance.

Philippe et al. (2023) validated the Italian version of the scale. They conducted an EFA using Principal Axis Factoring with Oblimin rotation, analyzing a sample of 419 undergraduate and postgraduate students majoring in music performance-related disciplines from Italian music conservatories. Five factors were extracted: Music Performance Anxiety Symptoms (21 items), Depression and Hopelessness (6 items), Parental Support (3 items), Memory Self-Efficacy (2 items), and Generational Transmission of Anxiety (2 items). The initial EFA model explained 45.8% of the variance.

Barros et al. (2024) developed and validated the Portuguese Music Performance Anxiety Scale (PoMPAS) using a combination of EFA and CFA. The EFA employed Robust Diagonally Weighted Least Squares (RDWLS) with Robust Promin oblique rotation, while CFA utilized Diagonally Weighted Least Squares (DWLS). The study analyzed a sample of 414 Portuguese higher music education students, representing diverse instrumental backgrounds: Woodwinds (26.1%), Brass (21.5%), Frictional Strings (21.7%, e.g., violins, cellos), Keyboard Instruments (13.8%), Voice (7.2%), Finger Strings (6.8%), Percussion (1.7%), Electric Instruments (0.2%), and Conducting (1.0%). Three distinct factors emerged from the EFA: a Behavioral/Emotional Factor (12 items), a Contextual/Physiological Factor (10 items), and a Cognitive Factor (5 items), collectively explaining 52.64% of the variance. The subsequent CFA confirmed the structural validity of the three-factor model, demonstrating a strong fit index.

Takagi et al. (2025) validated the Japanese version of the scale. Employing EFA with Maximum Likelihood estimation and Promax rotation, they analyzed a sample of 400 Japanese musicians, comprising 77.25% instrumentalists and 22.75% vocalists. Seven factors were extracted: Music Performance Anxiety Symptoms (10 items), Psychological Vulnerability (8 items), Worry/Dread Focused on Self/Other Scrutiny and Evaluation (6 items), Parental Support (3 items), Memory and Self-Efficacy (3 items), Uncontrollability (2 items), and Generational Transmission of Anxiety (2 items). The seven-factor EFA model accounted for 55.8% of the variance.

Existing studies consistently confirm that MPA is a complex, multifaceted phenomenon encompassing physiological, emotional, behavioral, and cognitive factors. As a widely adopted assessment instrument, the K-MPAI-R has exhibited robust reliability and validity across diverse contexts. Nevertheless, cross-cultural validation studies have yielded inconsistent factor structures. These discrepancies suggest that cultural backgrounds not only shape the expression of MPA but also influence its conceptualization and measurement. Furthermore, prior research has rarely distinguished between vocal and instrumental performers, despite notable differences in their performance contexts and stressors. These research gaps highlight the need to reevaluate the K-MPAI-R within the specific context of Chinese university music students, particularly those specializing in vocal music and situated within the Confucian cultural framework.

## 3 Materials and methods

### 3.1 Research design and sampling

This research utilized a quantitative cross-sectional design, using a questionnaire as the main instrument for data collection. The participants were university students specializing in vocal music. All questionnaires were collected via an online questionnaire distribution and data collection platform named WJX (full name: Wenjuanxing), which is widely used in academic research and practical surveys in China. The questionnaires gathered in this study had no missing data since the researcher’s setting prohibited the submission of questionnaires with unanswered questions, except for the age, which is considered relatively sensitive personal information. This research used a two-step method combining judgmental and simple random sampling for data collection. Judgmental sampling was carried out through screening questions, with only vocal music major undergraduates being eligible to participate. After that, to ensure the randomness of data extraction, the researchers asked eight faculty members from those three universities’ music departments to share the questionnaire’s URL and QR code in students’ social network group chats. Ultimately, 922 questionnaires were collected. Among them, 123 questionnaires with less than 2 min’ response times and 63 with overly consistent answers were eliminated. As a result, 736 questionnaires were deemed valid, achieving a valid rate of 79.83%, with gender distribution showing 362 males (49.2%) and 374 females (50.8%). Of the 736 participants, 462 reported their age, with a mean of 20.28 and a standard deviation of 1.528.

To ensure the robustness of the factor solution, independent validation, and sample adequacy, the full sample (*N* = 736) was randomly and evenly divided into two subsamples (*n* = 368 each) using Excel’s RAND() function; an EFA was performed on Subsample 1 and a CFA on Subsample 2.

**FIGURE 1:**
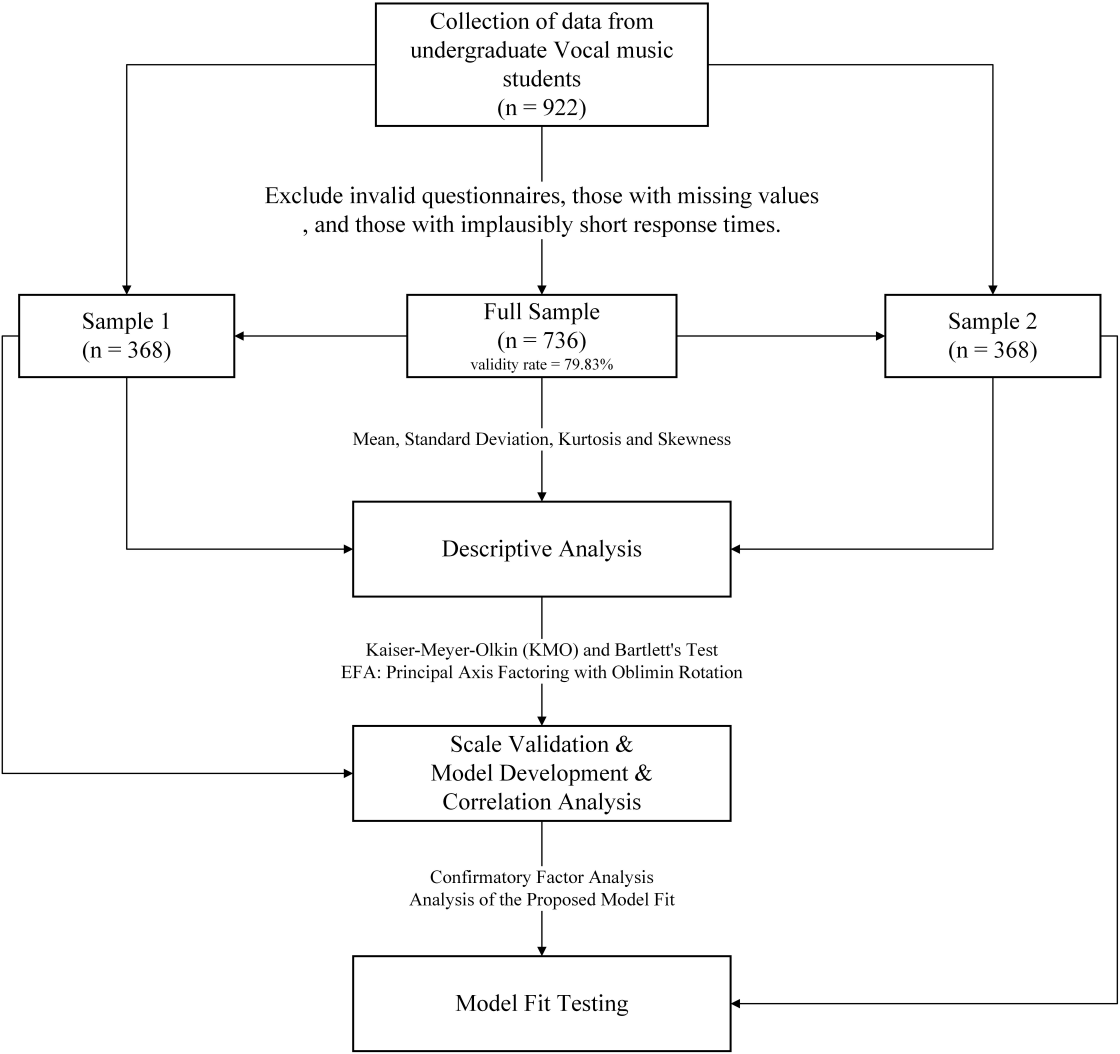
Participant flow and analysis pipeline.

As shown in Figure 1, the researchers followed the research process outlined below. Descriptive analyses of demographic variables (gender and age) and each scale item were performed using SPSS 27. For Sample 1, exploratory factor analysis (EFA) was conducted: KMO and Bartlett’s tests confirmed data suitability, and principal axis factoring with oblimin rotation was applied to extract factors and items and to propose an EFA model. AMOS 27 was then used to run confirmatory factor analysis (CFA) on Sample 2 to evaluate model fit.

### 3.2 Research instruments

The questionnaire used in this study was the K-MPAI-R – a widely used tool for assessing MPA, revised by Kenny (2009) based on Barlow’s emotional theory of anxiety disorders. Derived from the original 26-item K-MPAI (Kenny et al., 2004), the revised version expands to 40 items to comprehensively evaluate the emotional, physiological, cognitive, and behavioral factors of MPA while exploring underlying psychological developmental pathways (Kenny, 2011).

The researchers reached out to Professor Kenny via email, seeking permission to use this questionnaire. With great kindness, Professor Kenny granted permission and provided a Chinese language and certified questionnaire version. The researchers utilized a seven-point Likert scale ranging from 1 (strongly disagree) to 7 (strongly agree) for K-MPAI-R. It is worth highlighting that the K-MPAI-R incorporates several reverse-worded questions, including items 1, 2, 9, 17, 23, 33, 35, and 37, which deviate from the other inquiries regarding MPA. The researchers chose not to modify these questions. Instead, their values were reversed during the data analysis stage.

During the data analysis phase, the researchers first evenly and randomly divided the full sample into two subsamples, where Sample 1 contained 368 participants, while Sample 2 contained 368 participants.

## 4 Results

### 4.1 Descriptive analysis

Before conducting the factor analysis, the researchers utilized SPSS 27 software to analyze the mean, standard deviation, skewness, and kurtosis of each item in the K-MPAI-R under the full sample, and the results are shown in Table 1. Whenever the skewness and kurtosis of the data fall between the range of −2 to +2, the dataset can be considered as nearly normally distributed (Mallery and George, 2005). Table 2 indicates that the skewness for all items ranges from −0.287 to 0.216, while the skewness ranges from −1.012 to −0.120. Consequently, the data are approximately normally distributed for all items, making them appropriate for subsequent analysis.

**TABLE 1:** Mean, SD, skewness, kurtosis of K-MPAI-R items.

Item	Mean	SD	Skewness	Kurtosis
MPA1	3.250	1.059	−0.105	−0.533
MPA2	3.100	1.244	−0.011	−1.000
MPA3	4.090	1.077	0.216	−0.198
MPA4	4.100	1.198	−0.037	−0.840
MPA5	3.180	1.120	0.164	−0.879
MPA6	4.030	1.098	0.060	−0.442
MPA7	4.150	1.137	0.184	−0.349
MPA8	3.780	1.466	0.060	−1.000
MPA9	2.980	1.038	0.016	−0.559
MPA10	4.310	1.162	0.081	−0.538
MPA11	4.270	1.120	0.068	−0.535
MPA12	4.550	1.248	0.048	−0.473
MPA13	3.740	1.140	0.059	−0.379
MPA14	4.330	1.060	−0.004	−0.324
MPA15	4.520	1.122	0.135	−0.549
MPA16	4.280	1.086	0.116	−0.480
MPA17	3.190	1.060	−0.081	−0.508
MPA18	4.660	0.992	0.003	−0.348
MPA19	4.060	1.087	0.024	−0.199
MPA20	4.040	1.063	0.162	−0.309
MPA21	4.140	1.314	−0.029	−1.012
MPA22	4.680	1.067	−0.065	−0.309
MPA23	3.150	0.982	−0.153	-−0.342
MPA24	4.140	1.041	0.043	−0.243
MPA25	4.490	1.037	0.073	−0.215
MPA26	4.240	1.149	0.078	−0.552
MPA27	3.420	1.194	0.048	−0.994
MPA28	4.070	1.017	0.122	−0.120
MPA29	3.760	1.225	0.049	−0.764
MPA30	4.630	1.050	0.147	−0.329
MPA31	4.210	1.029	0.144	−0.126
MPA32	4.170	1.283	−0.046	−0.949
MPA33	3.180	0.948	−0.287	−0.241
MPA34	4.130	1.037	0.085	−0.135
MPA35	3.210	1.112	−0.157	−0.610
MPA36	3.730	1.077	0.112	−0.487
MPA37	3.150	1.103	−0.115	−0.699
MPA38	4.340	1.033	0.029	−0.290
MPA39	4.190	1.147	−0.070	−0.203
MPA40	3.900	1.233	0.099	−0.748

**TABLE 2:** Kaiser-Meyer-Olkin (KMO) and Bartlett’s test for suitability of K-MPAI-R item data.

KMO and Bartlett’s test		
Kaiser-Meyer-Olkin measure of sampling adequacy.		0.967
Bartlett’s test of sphericity	Approx. Chi-Square	8504.291
	df	780
	Sig.	<0.001

### 4.2 Exploratory factor analysis (EFA)

The EFA was based on the data from Sample 1. Prior to formally commencing the factor analysis, the researchers performed the Kaiser-Meyer-Olkin (KMO) test and Bartlett’s test on all items. The KMO test assesses sampling adequacy, with a score over 0.9, indicating that the data is excellent for factor analysis; While Bartlett’s test is employed to examine the correlations among variables, the data is considered suitable for factor analysis when its *p*-value is less than 0.05 (Kaiser, 1974). As shown in Table 2, the KMO value is 0.967, whereas Bartlett’s Test of Sphericity results proved a significant correlation (*p* = 0.000 < 0.05). Therefore, sample 1 is suitable for the next EFA.

**FIGURE 2:**
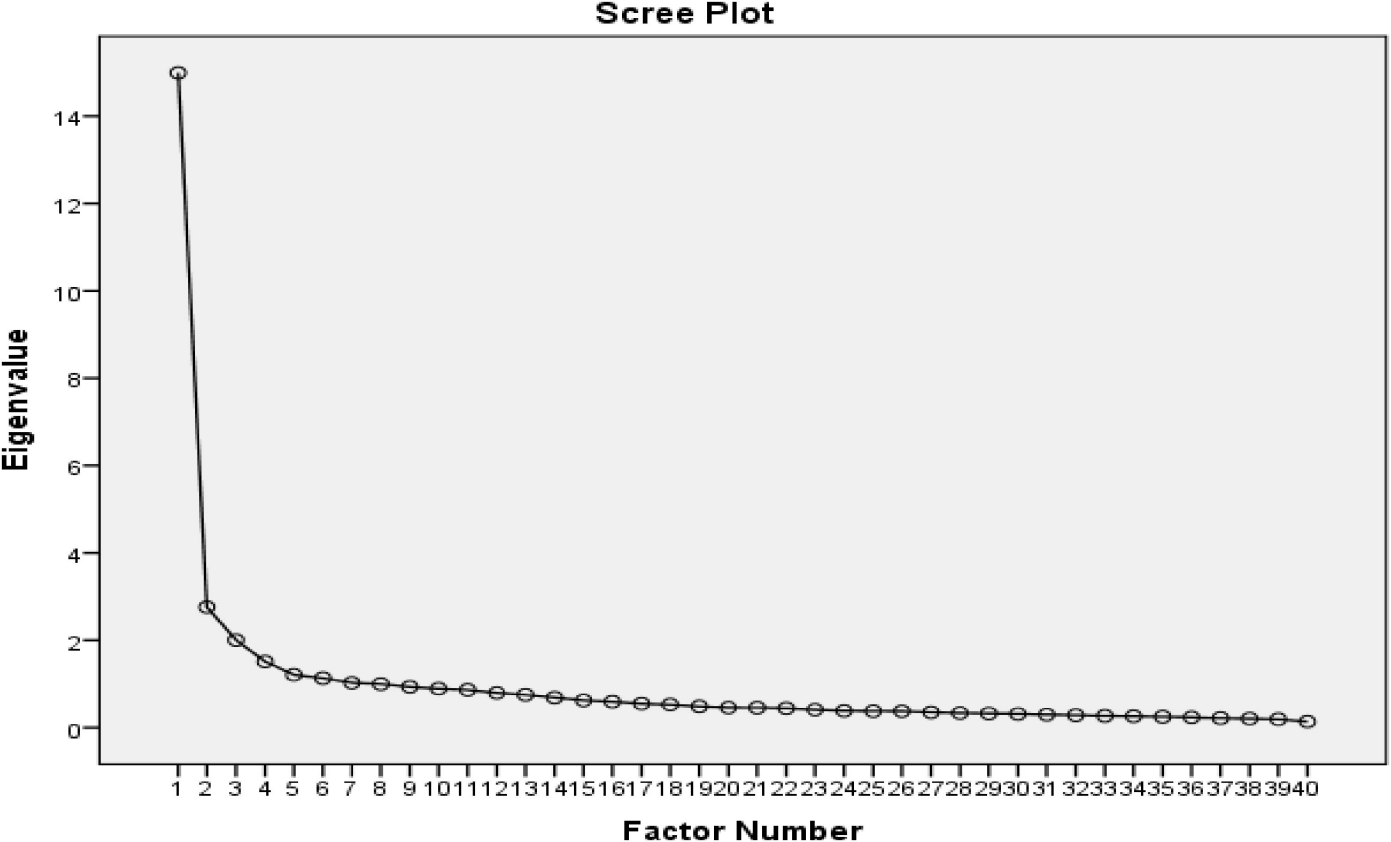
Scree plot for K-MPAI-R exploratory factor analysis.

The researchers used the principal axis factoring method with oblimin rotation to formally conduct the EFA. Four factors with eigenvalues greater than one were extracted, and the cumulative variance explained was 53.20%. The Scree Plot is shown in Figure 2. Additionally, the Cronbach coefficients for these four factors ranged from 0.740 to 0.921, indicating that the factors were sufficiently internally consistent. Items with loadings above 0.4 are considered stable and significant and should be associated with only one factor rather than two or more (Guadagnoli and Velicer, 1988). The researchers excluded Items 2, 4, 5, 8, 21, 27, 29, 32, 39, and 40 because their factor loadings were below 0.4 in all factors. Additionally, Items 14, 17, 24, and 28 were excluded as they simultaneously had factor loadings higher than 0.4 in two factors. Ultimately, the four factors with their corresponding items are shown in Table 3.

**TABLE 3:** Kenny Music Performance Anxiety Inventory-Revised (K-MPAI-R) exploratory factor analysis: Chinese vocal majors.

No.	Items	Factor			
		PV	PSCA	PS	MSE
MPA18	I am often concerned about a negative reaction from the audience.	0.767			
MPA20	From early in my music studies, I remember being anxious about performing.	0.729			
MPA1	I generally feel in control of my life (reversed).	0.722			
MPA38	I am concerned about being scrutinized by others.	0.722			
MPA3	Sometimes I feel depressed without knowing why.	0.711			
MPA6	I often feel that life has not much to offer me.	0.666			
MPA19	Sometimes I feel anxious for no particular reason.	0.652			
MPA34	I worry so much before a performance, I cannot sleep.	0.645			
MPA25	After the performance, I worry about whether I played well enough.	0.625			
MPA7	Even if I work hard in preparation for a performance, I am likely to make mistakes.	0.604			
MPA13	I often feel that I am not worth much as a person.	0.557			
MPA31	As a child, I often felt sad.	0.485			
MPA36	Prior to, or during a performance, I experience shaking or trembling or tremor.		0.773		
MPA16	Prior to, or during a performance, I feel sick or faint or have a churning in my stomach.		0.756		
MPA26	My worry and nervousness about my performance interferes with my focus and concentration.		0.755		
MPA22	Prior to, or during a performance, I experience increased heart rate like pounding in my chest.		0.733		
MPA11	I never know before a concert whether I will perform well.		0.699		
MPA30	Prior to, or during a performance, I have increased muscle tension.		0.667		
MPA15	Thinking about the evaluation, I may get interferes with my performance.		0.656		
MPA12	Prior to, or during a performance, I experience dry mouth.		0.615		
MPA10	Prior to, or during a performance, I get feelings akin to panic.		0.515		
MPA33	My parents encouraged me to try new things (reversed).			0.770	
MPA23	My parents always listened to me (reversed).			0.683	
MPA9	My parents were mostly responsive to my needs (reversed).			0.612	
MPA37	I am confident playing from memory (reversed).				0.876
MPA35	When performing without music, my memory is reliable (reversed).				0.680
No. of items		12	9	3	2
Cronbach’s alpha		0.873	0.921	0.748	0.740
% of variance		37.45%	6.90%	5.02%	3.79%
Total variance explained		53.20%			

Based on the factors’ names from previous related studies and the nature of the items included in each factor:

Factor 1: Psychological Vulnerability – PV (including items 1, 3, 6, 7, 13, 18, 19, 20, 25, 31, 34, and 38);

Factor 2: Proximal Somatic and Cognitive Anxiety – PSCA (including items 10, 11, 12, 15, 16, 22, 26, 30, and 36);

Factor 3: Parental Support – PS (including items 9, 23, and 33);

Factor 4: Memory Self-Efficacy – MSE (including items 35 and 37).

The Pearson correlation matrix for the four factors is presented in Table 4. Notably, to ensure directional consistency within the PV factor, Item 1 was again reverse-scored; all items comprising the PS and MSE factors were retained in their original scoring direction. All correlations were statistically significant at *p* < 0.001, with values ranging from −0.308 to 0.569.

**TABLE 4:** Pearson correlation matrix of four EFA-extracted factors.

Factors	PV	PSCA	PS	MSE
PV	1	0.569***	−0.388***	−0.360***
PSCA	0.569***	1	−0.322***	−0.378***
PS	−0.388***	−0.322***	1	0.308***
MSE	−0.360***	−0.378***	0.308***	1

***p < 0.001.

### 4.3 Confirmatory factor analysis (CFA)

Subsequently, the researchers conducted a CFA with data from Sample 2 using Amos 24. The fit indices for sample 2 in the proposed model are as follows: the CMIN/DF is 2.2246, the GFI is 0.8739, the AGFI is 0.8490, the CFI is 0.9441, the NFI is 0.9034, the TLI is 0.938, and the RMSEA is 0.0578. The fit metrics of the model fall within the defined acceptable range, and therefore, the measurement model is well-fitted, as indicated by previous studies (Byrne, 1994; Hooper et al., 2007; Schermelleh-Engel et al., 2003). Sample 2’s data showed a good fit within the model derived from Sample 1, indicating that the model’s stability was initially established. Furthermore, Cronbach’s alpha values ranged from 0.760 to 0.936, indicating that the factors had sufficient reliability and internal consistency.

## 5 Discussions

This study employed a “two-stage quantitative validation design” to validate the Chinese version of the K-MPAI-R. 736 Chinese undergraduate students majoring in vocal music were evenly divided into two subsamples (368 participants each). For one subsample, EFA was conducted using principal axis factoring with oblimin rotation, which yielded four factors: PV, PSCA, PS, and MSE, with a cumulative variance explanation rate of 53.20%. Meanwhile, CFA was performed on the other subsample. The results indicated a good model fit, indicating that the scale possesses satisfactory construct validity and internal consistency reliability among Chinese vocal music students.

A total of ten items (Items 2, 4, 5, 8, 21, 27, 29, 32, 39, and 40) were eliminated due to factor loadings below 0.40, while an additional four items (Items 14, 17, 24, and 28) were removed owing to cross-loadings above 0.40 on multiple factors. Although this procedure adheres to conventional psychometric standards, further explanations for the elimination of these items can be provided by contextualizing them within the cultural and sample-specific contexts. For instance, Items 2, 4, and 8 reflect broad personality traits (e.g., interpersonal trust, energy level) and exhibit weak relevance to the context-specific construct of music performance anxiety (MPA); Item 21, which centers on worries about “career failure,” lacks realistic salience for undergraduate students who have not yet entered professional pathways; Items 27, 5, and 29–pertaining to childhood emotional experiences and parental anxiety–may have induced inconsistent responses due to cultural sensitivity or memory ambiguity; Items 32 and 39 possess semantic ambiguity, which tends to simultaneously activate multiple responses related to self-evaluation, anxiety, and memory, thereby impairing factor convergence; and Item 40 may elicit socially desirable responses driven by social expectations in Confucian culture, leading to a skewed distribution. Regarding Items 14, 17, 24, and 28, their content typically encompasses multiple psychological dimensions (e.g., cognitive load, self-efficacy, avoidance behavior, and catastrophic thinking), rendering them prone to loading on multiple factors concurrently and undermining structural clarity. Overall, these findings suggest that in the cross-cultural application of the K-MPAI-R, beyond statistical indicators, the semantic clarity, cultural adaptability, and sample resonance of items are also critical factors influencing measurement validity.

Based on the context of Chinese Confucian culture (e.g., “face” culture, emotional suppression) and the high-exposure nature of vocal music performance (characterized by “using the body as an instrument”), this study further refined the structure into a four-factor model. This structure exhibits a high degree of consistency with the core components of the original model proposed by Kenny (2009). Specifically, PV corresponds with that of the original model, while PS corresponds to the original “Early Relationship Context” factor. Meanwhile, the original “Proximal Performance Concerns” factor was refined and reconceptualized into two independent factors: PSCA and MSE. Notably, in Kenny’s original model, Items 7 (“Even if I work hard to prepare for a performance, I am still likely to make mistakes”), 25 (“After a performance, I worry about whether I performed well enough”), 34 (“I cannot sleep before a performance”), and 38 (“I am concerned about being scrutinized by others”) were categorized under the “Proximal Performance Concerns” factor. However, in the present study, these items clustered under the PV. This factor migration phenomenon may reflect that when confronting the aforementioned performance pressures, Chinese vocal music students tend to attribute such pressures to uncertainties about their own abilities, sensitivity to failure, and excessive focus on external evaluations, thus manifesting deeper experiences of self-vulnerability, rather than merely situational anxiety tied to the immediate performance context. Furthermore, the present study observed that Item 11 (“I never know before a concert whether I will perform well”) was classified under the PV factor in Kenny’s original model, but was reassigned to PSCA in this study. This shift may stem from the body-as-instrument nature of vocal performance: students are more inclined to interpret “uncertainty about performance quality” as issues related to state fluctuations on the day of the performance, compromised bodily control, or difficulties in attention regulation, thus attributing it to situational anxiety responses rather than stable vulnerabilities in self-structure. This reorganization of factor structure suggests that the underlying psychological mechanisms of MPA may exhibit variability across different cultural and professional contexts. It also emphasizes that MPA should be understood as a multidimensional psychological phenomenon shaped by culture, self-construal, and social evaluation systems.

The first core factor extracted in this study is PV, encompassing 12 items (1, 3, 6, 7, 13, 18, 19, 20, 25, 31, 34, 38). It is primarily characterized by high sensitivity to failure, criticism, and negative evaluations, coupled with low self-esteem and insufficient self-efficacy. This factor has also been validated in the French version (Philippe et al., 2022) and the Japanese version (Takagi et al., 2025) of the K-MPAI-R scale. However, there are subtle differences in the focus of this factor across versions from different cultures. The French version, which includes items 1, 3, 4, 6, 13, 19, 24, 27, and 31, places greater emphasis on “intrinsic uncertainty” and “feelings of self-failure.” In contrast, the structure revealed in the present study indicates that individuals’ psychological vulnerability stems more from external evaluative pressure and the shame associated with “failure to meet social expectations.” This discrepancy may be attributed to the construct of “Interdependent Self-Construal” in the Confucian cultural context, where individuals’ self-evaluations are highly dependent on the assessments of authorities or others, leading to a rapid elevation in anxiety levels when confronted with public evaluation scenarios (Markus and Kitayama, 1991). Notably, while the present study differs from the Japanese version (Takagi et al., 2025) in the item composition of the PV factor (e.g., the Japanese version includes items 3, 4, 6, 8, 13, 19, 20, 31), the two studies maintain a high degree of consistency in their core construct. The overlapping items reflect the underlying psychological mechanisms contributing to the development of MPA, such as difficulties in emotion regulation (item 3), negative self-evaluation (items 13, 19, 20, 31), and personality vulnerability (items 6, 31). These characteristics are highly consistent with the description of highly susceptible individuals in Barlow’s (2000) emotional disorder theory, which emphasizes that individuals tend to experience cognitive-affective vulnerability when facing performance situations with high evaluative pressure. These findings also align with Hofmann et al. (2010) and Marques et al. (2011), who found that in East Asian cultural contexts, psychological vulnerability (encompassing difficulties in emotion regulation, negative self-evaluation, and personality vulnerability) may act as a stable risk factor influencing the onset of anxiety, which also applies to MPA. Besides, there are differences between the present study and the Japanese version study in terms of sample composition (e.g., the present study focuses on undergraduate vocal music students, while the Japanese study uses a mixed sample), language translation details, and factor extraction criteria. Therefore, the exclusion or inclusion of certain items in the two studies more reflects micro-level differences in linguistic semantic adaptability, sample composition structure, or psychological response patterns, rather than systematic differences at the macro-cultural dimension. This suggests that in cross-cultural validation studies, greater emphasis should be placed on construct consistency and the inherent commonality of psychological structures, while avoiding overinterpretation of item differences. Furthermore, in the Spanish validation study by Alzugaray et al. (2016), a “Helplessness” factor was extracted (items 1, 3, 4, 5, 6, 11, 16, 21, 23, and 26). Despite the difference in factor naming compared to the “Psychological Vulnerability” construct identified in the present study, the content of the items reflects similar psychological mechanisms, including low self-efficacy, fear of failure, and high sensitivity to negative evaluations. Thus, from the perspective of construct validity, the “Helplessness” factor can be regarded as a culturally adaptive expression of psychological vulnerability.

In the present study, PSCA extracted nine items, which reflect the high level of tension vocal students feel before or during a performance, as evidenced by increased heart rate, muscle tension, disturbed thinking, and pessimistic predictions. In the Barros et al.’s (2024) Portuguese version, the PSCA split into two separate factors: somatic anxiety and cognitive anxiety. By contrast, the PCSA in this study emerged as a single unified factor, a difference that may stem from Confucian cultural traits such as “face-saving” and “emotional suppression” where students often avoid direct expression of anxiety or help-seeking behaviors, thereby increasing the likelihood of cognitive stress manifesting as physical symptoms and triggering a cascade of physiological stress reactions that form an integrated anxiety pattern encompassing both cognitive and somatic features. PSCA was also extracted in the French version, which contains 11 items, but the content is more oriented toward the processes involved in the onset of performance-related anxiety (Philippe et al., 2022). In comparison with the Japanese version, which is also influenced by Confucian culture (Takagi et al., 2025), the factors in this study are more inclined to reflect the physical and mental anxiety responses in performance situations, encompassing two components: somatic reactions during performance (such as trembling) and cognitive-level uncertainties (such as ambiguity regarding performance quality), this may be related to the fact that the vocal performer’s “body is a musical instrument.” Vocal performers must engage their entire body in the vocalization process, encompassing breath control, resonance management, facial expression, and posture coordination, among other elements, all of which are prone to “losing control” in high-pressure situations, where even a minor mistake can instantly expose emotional states and trigger a chain reaction of technical breakdowns. The “unconcealable” performance style places extremely high demands on performers’ sense of control over details, resulting in more concentrated and intense cognitive and somatic anxiety symptoms among vocal performers.

The third factor extracted in this study is PS, encompassing Items 9, 23, and 33. This factor has been consistently identified in structural validation studies across multiple language versions, demonstrating favorable cross-cultural construct stability. In the Romanian version (Faur et al., 2021), Portuguese version (Dias et al., 2022), Italian version (Philippe et al., 2023), and Japanese version (Takagi et al., 2025), the structure and content of this factor exhibit a high degree of consistency. Meanwhile, in the Spanish version by Alzugaray et al. (2016), the Brazilian Portuguese version by Barbar et al. (2015), and the French version by Philippe et al. (2022), although labeled as “Early Relationship Context,” the core construct still focuses on supportive experiences within early family relationships. Such cross-linguistic consistency indicates that early family contexts largely influence the development of MPA. Across various cultural settings, parents typically engage highly in their children’s music learning processes, with their encouragement, expectations, and even controlling behaviors collectively shaping the developmental trajectories of music-major students. Notably, parental overstimulation and excessive expectations may also serve as potential sources of MPA.

The fourth factor extracted in this study is MSE, encompassing Items 35 and 37, which primarily measures individuals’ confidence in and ability to recall musical material during performances accurately. This factor has been consistently identified in structural validation studies across multiple language versions, demonstrating robust cross-cultural construct stability, such as in the Romanian version (Faur et al., 2021), Portuguese version (Dias et al., 2022), Italian version (Philippe et al., 2023), and Japanese version (Takagi et al., 2025). In the study on the French version (Philippe et al., 2022), although this factor was labeled “Confidence in Memory,” its core construct remains consistent, focusing on performers’ concerns about memory errors and their sense of control over memory.

Across cross-cultural studies on the K-MPAI-R among various language versions, the number of extracted factors ranges from 3 to 7 due to differences in cultural contexts and sample compositions. Examples include Portuguese versions with a 3-factor structure (Barbar et al., 2015; Barros et al., 2024), Spanish versions (Alzugaray et al., 2016; Chang-Arana et al., 2018), Portuguese (Dias et al., 2022) and Romanian (Faur et al., 2021) versions with a 4-factor structure, Italian (Philippe et al., 2023) and French (Philippe et al., 2022) versions with a 5-factor structure, and a Japanese version (Takagi et al., 2025) with a 7-factor structure, which reflects the adaptive manifestation of anxiety structures across different cultures. Notably, at least one factor focusing on the core symptoms of MPA has been consistently identified in existing studies, such as “Worries and Insecurity” (Barbar et al., 2015), “Specific Cognitions” (Alzugaray et al., 2016), “Proximal Somatic and Cognitive Anxiety” (Philippe et al., 2022), “Contextual/Physiological Factor” (Barros et al., 2024), “Music Performance Anxiety” (Chang-Arana et al., 2018), and “Music Performance Anxiety Symptoms” (Faur et al., 2021; Dias et al., 2022; Philippe et al., 2023; Takagi et al., 2025), which demonstrates that the K-MPAI-R possesses cross-cultural construct stability at the level of anxiety symptoms.

This study focuses on university students majoring in vocal music, and the professional characteristics of vocal performers may have influenced the structural composition of MPA-related factors to some extent. PV in this study reflects the deep-seated psychological traits of vocal performers, including difficulties in emotional regulation, personality-based insecurity, and negative self-evaluation. The existence of PV is closely linked to the highly self-exposed nature of vocal performance, as vocal performers rely not only on their bodies as direct vocalization media but also need to establish emotional connections with the audience through facial expressions and emotional conveyance. Consequently, they are highly susceptible to the dual pressures of social evaluation and self-denial. In mixed-sample studies, factors related to negative emotions have also been identified. For example, studies by Faur et al. (2021) and Dias et al. (2022), in which vocal music majors accounted for 15.95% and 9.1% respectively, both extracted a “Depression and Hopelessness” factor. However, the interpretive scope of PV in these two aforementioned studies is relatively limited, primarily manifesting as negative emotions and hopelessness, and is unable to adequately uncover personality-level insecurity and self-denial experiences associated with social exposure. PV extracted in the study by Takagi et al. (2025), where vocal music majors constituted 22.75%, is more consistent with that of the present study. Specifically, Takagi et al.’s (2025) PV factor not only includes negative emotional experiences such as depression, anxiety, and hopelessness but also encompasses difficulties in depending on others (Item 8) and feelings of anxiety during early developmental stages (Item 20), albeit with a relatively small number of relevant items. This may imply that as the proportion of vocal music students in mixed samples increases, it becomes more likely to identify dimensions of Psychological Vulnerability that are congruent with the traits of vocal performers. Barbar et al. (2015) employed the 26-item version of the scale developed by Kenny in 2004 (with vocal music majors accounting for 41.6%) and extracted two anxiety-related factors, “Depression and Hopelessness” and “Worries and Insecurity.” Among them, the “Depression and Hopelessness” factor overlaps partially with the PV factor in the present study, both centering on experiences such as low mood, anxiety, and helplessness. Similarly, in a study focusing on instrumental samples, Alzugaray et al. (2016) also used the 26-item version and extracted a “Helplessness” factor, which similarly focuses on negative emotion and lack of sense of control. This may be attributed to the fact that the item coverage of the 26-item version is inherently relatively limited, failing to encompass the content directly related to social evaluation and self-negation identified in the present study. In contrast, the 40-item version of the scale is more comprehensive in terms of its theoretical framework and measurement dimensions, thereby enabling the current study to identify a more complex psychological vulnerability structure.

Proximal Somatic and Cognitive Anxiety extracted in this study reflects the immediate anxiety experiences of vocal music students before and during performances. This factor fully captures the intense tension felt by vocal performers in high-visibility performance settings, and this kind of tension extends beyond mere cognitive worries to be accompanied by distinct physiological arousal. In studies using mixed samples such as Faur et al. (2021), Dias et al. (2022), and Takagi et al. (2025), while similar items have been extracted, they are typically aggregated into the broader factor of “music performance anxiety symptoms.” Although the items included in these studies exhibit substantial overlap with the PSCA in the present study, these studies did not further distinguish between cognitive and somatic anxiety responses, instead interpreting them as a single integrated construct. In contrast, the present study aggregates cognitive and somatic anxiety responses into the independent PSCA factor and indicates the more concentrated and prominent nature of immediate tension experienced by vocal students during stage performances. This finding aligns closely with the professional characteristics of vocal performance: Vocalists must use their bodies directly as “instruments” for sound production, while simultaneously engaging in emotional communication with the audience through facial expressions and body movements. This performance mode renders them more prone to experiencing intense anxiety responses simultaneously at both the psychological (cognitive) and physiological (somatic) levels. PS was extracted in this study and has also been consistently supported in studies with mixed-discipline samples (Barbar et al., 2015, 26-item version; Faur et al., 2021; Dias et al., 2022; Takagi et al., 2025) as well as in studies with pure instrumental samples (Alzugaray et al., 2016), indicating the stability of this factor across different professional groups. Meanwhile, MSE in this study was also extracted in 26-item version mixed-sample K-MPAI-R revalidation studies by Faur et al. (2021), Dias et al. (2022), and Takagi et al. (2025), but it did not appear in the 26-item version studies by Barbar et al. (2015) and Alzugaray et al. (2016), primarily due to the absence of relevant items in the early 26-item version. This indicates that PS is a stable factor influencing MPA, consistently identified in studies involving vocal, instrumental, and mixed-major groups; whereas the detection of MSE is associated with the scale version, as it failed to emerge in some studies due to the early 26-item version lacking relevant items.

The Pearson correlation analysis provides additional insights into the underlying mechanism of MPA among Chinese vocal music majors. First, a moderate to strong positive correlation was observed between PV and PSCA. This finding aligns with Philippe et al. (2022)’s study and the multidimensional nature of MPA, suggesting that individuals with higher psychological vulnerability are more likely to experience somatic symptoms (e.g., trembling, increased heart rate) and cognitive disturbances (e.g., poor concentration, performance uncertainty) in high-stakes performance contexts. For vocal music students, who rely on their bodies as “instruments” and face direct audience scrutiny, this link may be particularly pronounced: psychological fragility could amplify the perception of performance pressure, triggering a cascade of physical and cognitive anxiety responses. Second, PS exhibited significant negative correlations with both PV and PSCA. This supports the protective role of parental involvement in mitigating MPA-related psychological distress. In the Confucian cultural context, where family approval is highly valued, supportive parental attitudes (e.g., active listening, encouragement) may enhance students’ self-worth and reduce their fear of negative evaluation, thereby alleviating both underlying psychological vulnerability and situational somatic-cognitive anxiety. Conversely, this correlation also implies that insufficient or overly demanding parental involvement could exacerbate MPA. Third, MSE showed significant negative correlations with PV and PSCA. This indicates that students with higher confidence in their ability to recall musical material accurately tend to report lower psychological vulnerability and fewer performance-related anxiety symptoms. Given that vocal performances often require off-score execution, confidence in memory may act as a buffer against performance pressure: reduced worry about memory failure could diminish self-doubt (a core feature of PV) and prevent the onset of somatic tension or cognitive distraction. This finding reinforces the importance of memory training and efficacy-building in MPA interventions for vocalists. Finally, a weak to moderate positive correlation was found between PS and MSE. This suggests that supportive parental environments may indirectly foster students’ memory self-efficacy, potentially by reducing performance-related stress that impairs memory retrieval, or by reinforcing confidence through consistent encouragement during practice. This indirect pathway highlights the interconnectedness of social support and individual cognitive resources in shaping MPA experiences. Collectively, these correlations confirm that isolated factors do not drive MPA among Chinese vocal music students, but rather through dynamic interactions between psychological vulnerability, situational anxiety responses, social support, and cognitive efficacy. The observed patterns underscore the need for integrated MPA interventions that address both individual psychological traits (e.g., building self-esteem and memory confidence) and social support systems (e.g., guiding parental support practices).

### 5.1 Implications

On a theoretical level, this study further re-validates the cross-cultural structural stability of K-MPAI-R in the core symptom dimensions of MPA, thereby providing further support for its potential applicability as an MPA assessment tool across diverse cultural contexts. On the other hand, by integrating the high-visibility characteristics of vocal performance with the social norms of Confucian culture (which emphasizes social evaluation and shame emotions), the study reveals how cultural and disciplinary factors collectively shape the factor structure of MPA. It highlights that MPA, as a multidimensional psychological experience, is dually influenced by cultural values and professional attributes. This finding expands the theoretical boundaries of existing MPA models and provides new empirical evidence for constructing an explanatory framework that integrates cultural differences and disciplinary specificities.

On a practical level, this study offers targeted recommendations for designing diversified MPA intervention pathways. For students, the extraction of MSE indicates that vocal music students are prone to anxiety triggered by concerns about forgetting lyrics, musical scores, or performance details when facing performances without sheet music or high-load memory tasks. Therefore, efforts should be made to enhance their cognitive regulatory efficacy in high-pressure performance contexts by training them in memory strategies, attention control, and on-the-spot adaptability. For parents, the stability of PS demonstrates that family upbringing styles have profound implications for students’ MPA. Rooted in the Confucian cultural context, Chinese university students (even when they have reached adulthood) still maintain close emotional and developmental bonds with their families. It is recommended that universities and teachers guide parents to improve their emotional support capabilities, refine communication approaches, and adjust unreasonable achievement expectations through family education workshops, school-family cooperation mechanisms, and other initiatives, thereby establishing a positive emotional support system at the family level. For music educators, the significant presence of the PV and PCSA suggests that educators need to enhance their awareness of identifying and intervening in students’ MPA manifestations. Educators should pay attention to students’ emotional and physical responses when receiving criticism, preparing for performances, and handling unexpected situations. They should also foster a safe and accepting teaching environment through positive feedback, stress-reducing rehearsal environments, and moderate evaluation mechanisms. For university administrators, there is a need to systematically integrate curriculum design, psychological counseling, and family collaboration mechanisms to establish a cross-departmental support system. On the basis of traditional skill training, music education curricula should incorporate modules on psychological resilience training, emotion regulation strategies, and performance psychology techniques, helping students improve their adaptability to MPA.

### 5.2 Limitations

This study has several limitations. First, the sample was limited to Chinese undergraduate vocal music students (18–24 years old), lacking diversity in age, skill level, and career stage (e.g., novice learners, professionals). This restriction limits the generalizability of the findings and precludes examination of the moderating effect of performance experience on the factor structure of MPA. Second, MPA was measured in general learning and performance contexts without differentiating among task types (e.g., exams, concerts, competitions), thereby overlooking situational variations in the triggering mechanisms and factor expressions of MPA. Third, the study relied exclusively on self-report measures, which are susceptible to social desirability bias–particularly in Confucian cultural contexts that emphasize emotional suppression and “face-saving,” and did not include complementary objective indicators (e.g., physiological measures, behavioral observations) to strengthen data robustness. Future research should address these limitations by expanding sample diversity, incorporating situational variables, and adopting mixed-methods designs.

## 6 Conclusion

Music performance anxiety (MPA) is a complex psychological phenomenon that significantly affects performers’ well-being and career development; yet, the assessment instruments frequently lack cultural and disciplinary specificity, especially for vocalists in non-Western settings. This study validates the cultural applicability of the K-MPAI-R among Chinese undergraduate students majoring in vocal music. While this population has received limited attention in existing literature, they exhibit unique anxiety vulnerability in high-visibility performance contexts. Through exploratory and confirmatory factor analyses, this study established a four-factor structure: Psychological Vulnerability, Proximal Somatic and Cognitive Anxiety, Parental Support, and Memory Self-Efficacy. These results reflect the multifaceted structural characteristics of MPA within the Confucian cultural context. The findings of this study not only verify the cross-cultural structural stability of the K-MPAI-R but also highlight context-specific aspects. For instance, the significance of “Parental Support” and “Memory Self-Efficacy” reflects the important role of external evaluation pressure and memory anxiety among Chinese vocal performers. Furthermore, the integration of somatic anxiety and cognitive anxiety into a single factor reveals that emotional suppression and fear of shame may induce more intense physiological stress responses, particularly in high-visibility vocal performance, where the body itself serves as the instrument. Theoretically, by integrating aspects of culture, social relationships, and professional characteristics, this study enriches and expands the disciplinary adaptability of existing MPA models. It emphasizes that MPA is not merely a situational anxiety response but a complex psychological phenomenon jointly shaped by culture, social relationships, self-cognitive patterns, and professional traits. Practically, the validated Chinese version of the K-MPAI-R provides a culturally sensitive measurement tool with good reliability and validity for identifying performance anxiety risks among music majors in colleges and universities.

## Data Availability

The raw data supporting the conclusions of this article will be made available by the authors, without undue reservation.

## References

[B1] Alzugaray F. J. Z. Hernández S. O. López O. C. Gil B. M. (2016). Kenny music performance anxiety inventory: Confirmatory factor analysis of the Spanish version. *Psychol. Music* 44 340–352. 10.1177/0305735614567932

[B2] Barbar A. E. M. Souza J. A. D. Osório F. D. L. (2015). Exploratory factor analysis of Kenny music performance anxiety inventory (K-MPAI) in a Brazilian musician sample. *Arch. Clin. Psychiatry* 42 113–116. 10.1590/0101-60830000000060

[B3] Barlow D. H. (2000). Unraveling the mysteries of anxiety and its disorders from the perspective of emotion theory. *Am. Psychol.* 55 1247–1263. 10.1037//0003-066x.55.11.1247 11280938

[B4] Barros S. França A. Marinho H. Pereira A. (2024). Music performance anxiety: Development and validation of the Portuguese music performance anxiety scale. *Front. Psychol.* 15:1436216. 10.3389/fpsyg.2024.1436216 39070583 PMC11276725

[B5] Bascomb J. S. (2019). *Performing arts and performance anxiety.* Theses, Dissertations and Capstones. Marshall University: Huntington, WV.

[B6] Byrne B. M. (1994). *Structural equation modeling with EQS and EQS/Windows: Basic concepts, applications, and programming.* Newcastle upon Tyne: Sage.

[B7] Chang-Arana ÁM. Kenny D. T. Burga-León A. A. (2018). Validation of the Kenny music performance anxiety inventory (K-MPAI): A cross-cultural confirmation of its factorial structure. *Psychol. Music* 46 551–567. 10.1177/0305735617717618

[B8] Dias P. Veríssimo L. Figueiredo N. Oliveira-Silva P. Serra S. Coimbra D. (2022). Kenny music performance anxiety inventory: Contribution for the Portuguese Validation. *Behav. Sci.* 12:18. 10.3390/bs12020018 35200271 PMC8869159

[B9] Driskill K. (2012). *Symptoms, causes, and coping strategies for performance anxiety in singers: A synthesis of research.* Morgantown, WV: West Virginia University.

[B10] Faur A. L. Vaida S. Opre A. (2021). Kenny music performance anxiety inventory: Exploratory factor analysis of the Romanian version. *Psychol. Music* 49 777–788. 10.1177/0305735619896412

[B11] Fernholz I. Mumm J. L. M. Plag J. Noeres K. Rotter G. Willich S. N. (2019). Performance anxiety in professional musicians: A systematic review on prevalence, risk factors and clinical treatment effects. *Psychol. Med.* 49 2287–2306. 10.1017/S0033291719001910 31474244

[B12] Guadagnoli E. Velicer W. F. (1988). Relation of sample size to the stability of component patterns. *Psychol. Bull.* 103 265–275. 10.1037/0033-2909.103.2.265 3363047

[B13] Han K. H. (2016). The feeling of “Face” in confucian society: From a perspective of psychosocial equilibrium. *Front. Psychol.* 7:1055. 10.3389/fpsyg.2016.01055 27486411 PMC4949215

[B14] Hofmann S. G. Anu Asnaani M. A. Hinton D. E. (2010). Cultural aspects in social anxiety and social anxiety disorder. *Dep. Anxiety* 27 1117–1127. 10.1002/da.20759 21132847 PMC3075954

[B15] Hofstede G. Hofstede G. J. Minkov M. (2010). *Cultures and organizations: Software of the mind*, 3rd Edn. Columbus, OH: McGraw Hill LLC.

[B16] Hooper D. Coughlan J. Mullen M. (2007). Structural equation modeling: Guidelines for determining model fit. *Electronic J. Bus. Res. Methods* 6 53–60. 10.21427/D7CF7R

[B17] Huang L. L. (2016). Interpersonal harmony and conflict for Chinese people: A Yin-Yang perspective. *Front. Psychol.* 7:847. 10.3389/fpsyg.2016.00847 27375526 PMC4896957

[B18] Juncos D. G. Heinrichs G. A. Towle P. Duffy K. Grand S. M. Morgan M. C. (2017). Acceptance and commitment therapy for the treatment of music performance anxiety: A pilot study with student vocalists. *Front. Psychol.* 8:986. 10.3389/fpsyg.2017.00986 28674509 PMC5475388

[B19] Kaiser H. F. (1974). An index of factorial simplicity. *Psychometrika* 39 31–36. 10.1007/BF02291575

[B20] Kenny D. (2011). *The psychology of music performance anxiety.* Oxford: Oxford University Press.

[B21] Kenny D. T. (2009). “The factor structure of the revised Kenny music performance anxiety inventory,” in *International symposium on performance science*, (Utrecht: Association Européenne des Conservatoires), 37–41.

[B22] Kenny D. T. (2023). The Kenny music performance anxiety inventory (K-MPAI): Scale construction, cross-cultural validation, theoretical underpinnings, and diagnostic and therapeutic utility. *Front. Psychol.* 14:1143359. 10.3389/fpsyg.2023.1143359 37325731 PMC10262052

[B23] Kenny D. T. Davis P. Oates J. (2004). Music performance anxiety and occupational stress amongst opera chorus artists and their relationship with state and trait anxiety and perfectionism. *J. Anxiety Disord.* 18 757–777. 10.1016/j.janxdis.2003.09.004 15474851

[B24] Mallery P. George D. (2005). *SPSS for windows step by step: A simple guide and reference 13.0 update.* Boston, MA: Allyn and Bacon.

[B25] Markus H. R. Kitayama S. (1991). Culture and the self: Implications for cognition, emotion, and motivation. *Psychol. Rev.* 98 224–253. 10.1037/0033-295X.98.2.224

[B26] Markus H. R. Kitayama S. (2014). “Culture and the self: Implications for cognition, emotion, and motivation,” in *College student development and academic life*. Abingdon: Routledge, 264–293.

[B27] Marques L. Robinaugh D. J. LeBlanc N. J. Hinton D. (2011). Cross-cultural variations in the prevalence and presentation of anxiety disorders. *Exp. Rev. Neurotherapeut.* 11 313–322. 10.1586/ern.10.122 21306217

[B28] Matsumoto D. Yoo S. H. Nakagawa S. (2008). Culture, emotion regulation, and adjustment. *J. Personal. Soc. Psychol.* 94 925–937. 10.1037/0022-3514.94.6.925 18505309

[B29] Papageorgi I. Hallam S. Welch G. F. (2007). A conceptual framework for understanding musical performance anxiety. *Res. Stud. Music Educ.* 28 83–107. 10.1177/1321103X070280010207

[B30] Philippe R. A. Cruder C. Biasutti M. Crettaz von Roten F. (2023). The Kenny music performance anxiety inventory-revised (K-MPAI-R): Validation of the Italian version. *Psychol. Music* 51 565–578. 10.1177/03057356221101430

[B31] Philippe R. A. Kosirnik C. Klumb P. L. Guyon A. Gomez P. Crettaz von Roten F. (2022). The Kenny music performance anxiety inventory–revised (K-MPAI-R): Validation of the French version. *Psychol. Music* 50 389–402. 10.1177/03057356211002642

[B32] Ryan C. Andrews N. (2009). An investigation into the choral singer’s experience of music performance anxiety. *J. Res. Music Educ.* 57 108–126. 10.1177/0022429409336132

[B33] Salmon P. G. (1990). A psychological perspective on musical performance anxiety: A review of the literature. *Med. Problems Perform. Art.* 5 2–11.

[B34] Schermelleh-Engel K. Moosbrugger H. Müller H. (2003). Evaluating the fit of structural equation models: Tests of significance and descriptive goodness-of-fit measures. *Methods Psychol. Res. Online* 8 23–74. 10.23668/psycharchives.12784

[B35] Spahn C. (2015). Treatment and prevention of music performance anxiety. *Prog. Brain Res.* 217 129–140. 10.1016/bs.pbr.2014.11.024 25725913

[B36] Steptoe A. (2001). “Negative emotions in music making: The problem of performance anxiety,” in *Music and emotion: Theory and research*, eds Juslin P. N. Sloboda J. A. (Oxford: Oxford University Press), 291–307.

[B37] Takagi S. Yoshie M. Murai A. (2025). Validation of the Japanese version of the Kenny music performance anxiety inventory-revised. *Front. Psychol.* 16:1543958. 10.3389/fpsyg.2025.1543958 40606897 PMC12213870

[B38] Zane N. Yeh M. (2002). “The use of culturally-based variables in assessment: Studies on loss of face,” in *Asian American mental health: Assessment theories and methods*, eds Kurasaki K. S. Okazaki S. Sue S. (Berlin: Springer), 123–138.

